# Epidemiology of HTLV-1 in Neyshabour, Northeast of Iran

**Published:** 2011-06-01

**Authors:** M R Hedayati-Moghaddam, F Fathimoghadam, I Eftekharzadeh Mashhadi, L Soghandi, H R Bidkhori

**Affiliations:** 1 Research Center for HIV/AIDS, HTLV and Viral Hepatitis, Iranian Academic Center, Mashhad Branch, Mashhad, Iran; 2 Center of Pathological and Medical Diagnostic Services, Iranian Academic Center for Education, Culture and Research (ACECR), Mashhad Branch, Mashhad, Iran

**Keywords:** HTLV-1 infection, Epidemiology, Iran

## Abstract

**Background:**

HTLV-1 infection is endemic in Mashhad, Northeast of Iran. This study was designed to assess the epidemiology of HTLV-1 and potential risk factors in Neyshabour, Northeastern Iran.

**Methods:**

All those who referred to Iranian Academic Center for Education, Culture and Research (ACECR)- Mashhad Laboratory in Neyshabour for evaluation of HTLV-I by ELISA, were tested using WB if the ELISA result was positive. A questionnaire about risk factors of infection was completed for all cases.

**Results:**

HTLV-1 infection was positive in 7.2% (35/483) of the participants according to the results of enzymelinked immunosorbent assay (ELISA) and western blot (WB) tests. Infection was significantly associated with age, family size, income and blood transfusion. However, gender, education, birthplace, race, marital status, and history of surgery, dentistry, traditional cupping, and hospitalization showed no significant relation.

**Conclusion:**

It seems that HTLV-I infection is highly endemic in Neyshabour and it is seems that more effective treatment strategies are needed.

## Introduction

Human T Lymphotrophic Virus type I (HTLV-I), a Deltavirus member of Retroviridae family has a cosmopolitan distribution and currently more than 20 million people in the world are infected with this virus. [1] Prevalence rate of more than 1% in the general population, Caribbean Basin, Central Africa, and South Japan are assumed as an endemic area for HTLV-I.[2] Northeast of Iran, Mashhad, the capital of Razavi Khorasan Province, is endemic for HTLV with a prevalence of 3% among general population.[3] In 2002, screening by enzyme-linked immunosorbent assay (ELISA) test for evaluation of HTLV infection revealed a prevalence of 3.4% in general population of Neyshabour, as the third largest city of the province.[4] However, confirming of the results by western blot (WB) was not performed in that study, and the precise prevalence of HTLV infection in this city is not determined yet. This study aims to evaluate the prevalence of HTLV infection and the sociodemographic and other potential risk factors among those who referred to Iranian Academic Center for Education, Culture and Research (ACECR)-Mashhad Clinical Laboratory in the city of Neyshabour, Northeastern Iran for the evaluation of HTLV-I.

## Materials and Methods

From February to August 2009 in a non-random sampling and a cross-sectional descriptive-analytic study, all those who referred to ACECR-Mashhad Clinical Lab in Neyshabour, Northeastern Iran for the evaluation of HTLV-1 infection (n=511) were enrolled. After a thorough explanation of the research project and invitation to participate in the study, a questionnaire on demographic features and potential risk factors of the infection such as a history of blood transfusion, surgery, dentistry procedures, being hospitalized, traditional cupping, and tattooing was completed for each case. Also an informed consent was obtained from each participant. After testing the blood samples by ELISA (ELISA-4; Dia. Pro diagnostic Bioprobes, Italy), which was ordered by physicians of the city, WB analysis (HTLV blot 2.4 kit, MP diagnostic Ltd., Germany) was used for the confirmation of positive ELISA results. Criteria for HTLV-I sero-positivity in WB test was reactivity to GAG (p19 with or without p24) and two ENV (GD21 and rgp 46-I) proteins. Data were analyzed using SPSS software (Version 13.0, Chicago, IL, USA) by Chi-Square and t tests. P values <0.05 were considered statistically significant.

## Results

The mean age of participants was 37.4±15.4 years (Range: 5-84 years). Four hundred and four cases (79.1%) were female, 94.9% were at least once married, 86.7% were born in Razavi Khorasan Province and 96.6% had Fars ethnicity. 27.6% lived in a small family size (≤2) and 27.2% in a large family size (>5). Also 14.9% were illiterate, 36.4% had 1-8 years of education, 30.8% had 9-12 years of education, and 17.9% had an academic educational level. Among the participants, 12.9% were governmental employee, 17.6% non-governmental, 5.0% student, and 64.5% were unemployed. 60.6% had less than 300 US$ monthly income, 33.2%, 300-500 US$, and 6.3% more than 500 US$. Pregnancy was the main reason (28.4%, 145/511) for which the participants were referred to the lab for evaluation of HTLV-1 infection. Other reasons were the patient’s request (21.7%, 111/511), being candidate for an operation (9%, 46/511), presence of indicative symptoms in patients (7.4%, 38/511), a positive history of the infection in at least one of the family members (2.2%, 11/511), and other reasons (4.1%, 21/511). The reason for patient’s referral was not determined in 139 cases (27.2%). History of blood transfusion was noticed in 4.6%, surgery in 39.4%, traditional cupping in 8.0%, dentistry procedures in 18.9%, hospitalization in 31.5%, tattooing in 1.2%, and imprisonment in 0.6% of the participants. ELISA was positive in 62 (12.1%) and 3 patients (0.6%) had borderline results. Among these two groups, WB analysis was performed for 37 cases (28 did not agree to perform WB). The WB result was positive in 94.6% (35/37); however the result was indeterminate in one case. Overall prevalence of HTLV infection was 7.2% (35/483) based on ELISA and WB results. Infection was significantly correlated with age (p<0.001), family size (p=0.004), income (p<0.001) and history of blood transfusion (p=0.024) (Table 1 and Table 2). Age was the most important factor which had the highest impact on the infection (OR= 8.05).

**Table 1:** Comparison of demographic features of HTLV positive and negative cases

		**No.**	**Positive cases (%)**	**Odd Ratio**	**95% CI**	***P* value**
Age	<30	197	3 (1.5)	8.05	2.43-26.73	<0.001
	≥30	280	31 (11.1)			
Sex	Male	97	10 (10.3)	0.60	0.28-1.31	0.196
	Female	385	25 (6.5)			
Birth place	Khorasan Razavi	422	32 (7.6)	0.61	0.08-4.69	0.631
	Other provinces	21	1 (4.8)			
Race	Fars	407	29 (7.1)	0.93	0.12-7.33	0.946
	Others	15	1 (6.7)			
Marital status	Married	418	26 (6.2)	2.60	0.93-7.27	0.060
	Non-married	34	5 (14.7)			
Family size	≤2	114	2 (1.8)	6.42	1.51-27.37	0.004
	>2	282	29 (10.3)			
Education	0-8 years	223	21 (9.4)	0.50	0.24-1.07	0.070
	≥9 years	221	11 (5.0)			
Job	Employed/Retired	126	12 (9.5)	0.71	0.34-1.51	0.337
	Non-employed	286	20 (7.0)			
Income (Monthly)	<300 US $	224	7 (3.1)	5.22	2.15-12.60	<0.001
	≥300 US $	146	21 (14.4)			

**Table 2:** Comparison of risk factors of HTLV positive and negative cases

		**No.**	**Positive cases (%)**	**Odd Ratio**	**95% CI**	***P* value**
Reasons of referring to Lab	Screening a	189	2 (1.1)	15.06	3.50-64.75	<0.001
	Non-screening	173	24 (13.9)			
History of blood transfusion	Positive	31	4 (12.9)	3.50	1.11-11.08	0.024
	Negative	443	18 (4.1)			
History of Surgery	Positive	187	16 (8.6)	0.55	0.26-1.16	0.110
	Negative	286	14 (4.9)			
History of Dentistry	Positive	93	9 (9.7)	0.57	0.26-1.29	0.174
	Negative	380	22 (5.8)			
History of traditional cupping	Positive	38	1 (2.6)	2.64	0.35-19.96	0.328
	Negative	435	29 (6.7)			
History of Hospitalization	Positive	149	10 (6.7)	0.91	0.42-2.01	0.823
	Negative	324	20 (6.2)			

^a^ Screening included pregnancy and being candidate from operation.

## Discussion

Areas with sero-prevalence of more than 2% are categorized as high endemic regions.[5] The current study showed a high prevalence of HTLV-I infection in a non-random sample of Neyshabour population. The high seroprevalence of HTLV infection by ELISA test had been previously shown in some studies. In 2002, Farid et al. revealed that the HTLV prevalence was 3.4% in the general population of Neyshabour.[4] HTLV infection is mainly localized in northeast of Iran including Mashhad and neighbor cities, and studies in several other areas, which are predominantly carried on blood donors demonstrated a lower prevalence from 0.01% to 0.62%.[6][7][8] On the other hand, many studies have shown that HTLV infection in high risk groups such as multitransfused patients throughout the country is more prevalent (1%-6.8%).[6][8][9][10][11][12] High prevalence of HTLV-1 infection found in this study might be due to a possible higher risk of infection in this sample, because our cases were referred for evaluation of HTLV by physicians of the city. There was a correlation between referral reasons and HTLV-1 seropositivity. Pregnancy was the main reason for referral (28.4%). The prevalence of the infection among pregnant women in our study was 1.39% which was a little higher than the same prevalence among pregnant women in Mashhad (0.9%).[13] Nevertheless, screening of HTLV infection by ELISA is not included in the national program of pre-natal care, but physicians refer pregnant women for HTLV serology test. However, HTLV screening is performed regularly in the blood donors of Khorasan provinces (North, South and Razavi) according to the guidelines of “Iranian Blood Ttransfusion Organization”. [14] In our study, 9% of the participants were referred by a surgeon for the evaluation of HTLV infection prior to the surgical operations which seems to become, a common reason for referral. The results of our study showed that the prevalence of the infection in patients who had been referred by a physician for screening (surgery/pregnancy) is relatively low, so it would be more efficient to only refer high risk or suspected cases for the screening. In this study, the correlation between some of the demographic features including age, family size, and income and HTLV infection was significant. However, birthplace, race, marital status, job, and literacy level did not have a significant impact on the infection. Several other studies represented age to have correlation with the infection.[15],[16] In evaluation of risk factors for HTLV sero-positivity, Murphy et al. showed no association between HTLV-I seropositivity and educational attainment, income or occupation. [17] However, education and ethnic affiliations were shown to have a relation with the infection.[15][18][19] On the other hand, there are some studies, presenting exactly the opposite results. A study in Panama demonstrated that race and socioeconomic factors were not associated with infection.[20] Thus, it seems that several other factors such as cultural and other demographic differences probably affect these associations. None of the potential non-sociodemographic risk factors of the infection including history of surgery, dentistry procedures, traditional cupping and hospitalization was found to be associated with the infection. However, our study is in consistent with several investigations and showed that blood transfusion had a correlation with HTLV infection.[15][21][22] As a conclusion, high prevalence of HTLV-1 infection, which was clearly shown in the current study,not only approves the previous findings, but also necessitates more attention and planning for the control of the infection in the Northeast of Iran.
